# Pyroptosis associated with immune reconstruction failure in HIV-1- infected patients receiving antiretroviral therapy: a cross-sectional study

**DOI:** 10.1186/s12879-022-07818-0

**Published:** 2022-11-21

**Authors:** Xiaojie Lao, Xinyin Mei, Jun Zou, Qing Xiao, Qiuyue Ning, Xianli Xu, Chunlan Zhang, Lei Ji, Shengwei Deng, Bingyang Lu, Maowei Chen

**Affiliations:** 1 Department of Infectious Diseases, Beijing Ditan Hospital Capital Medical University, Beijing, 100015 China; 2 Guangxi Key Laboratory of AlDS Prevention and Treatment, Nanning, 530021 China; 3 Department of Infectious Diseases, Guangxi Medical University First Affiliated Hospital, Nanning, 530021 China; 4 AIDS Clinical Treatment Center, The Fourth People’s Hospital of Nanning, Nanning, 530023 China; 5 Department of Infectious Diseases, Wuming Hospital of Guangxi Medical University, Nanning, 530100 China; 6 Department of Infectious Diseases, Mashan People’s Hospital, Nanning, 530600 China

**Keywords:** Human immunodeficiency virus (HIV), Immune reconstitution, Pyroptosis, Caspase-1, Gasdermin D (GSDMD)

## Abstract

**Background:**

Highly active anti-retroviral therapy (HAART) can successfully suppress human immunodeficiency virus (HIV) viral replication and reconstruct immune function reconstruction in HIV-1-infected patients. However, about 15–30% of HIV-1-infected patients still fail to recover their CD4^+^ T cell counts after HAART treatment, which means immune reconstruction failure. Pyroptosis plays an important role in the death of CD4^+^ T cells in HIV-1- infected patients. The study aims to explore the association between the expression of pyroptosis in peripheral blood and immune function reconstruction in HIV-1- infected patients.

**Methods:**

One hundred thirty-five HIV-1-infected patients including immunological non-responders (INR) group, immunological responders (IR) group and normal immune function control (NC) group were analyzed. The expression of GSDMD and Caspase-1 in peripheral blood of HIV-1-infected patients were measured by qPCR. The concentrations of GSDMD, Caspase-1, IL-1β and IL-18 in the peripheral serum were quantified by ELISA. The associations between the expression of pyroptosis in peripheral blood and immune function reconstruction were analyzed using multivariate logistic models.

**Results:**

The relative expression of GSDMD mRNA and caspase-1 mRNA in peripheral blood, as well as the expression of IL-18 cytokine in the INR, were significantly higher than those in the IR and NC (*P* < 0.05). There was no significant difference in the expression of IL-1β cytokine (*P* > 0.05). Multivariate logistic analysis showed that the patients with baseline CD4^+^ T cell counts less than 100 cells/μL (aOR 7.051, 95% CI 1.115–44.592, P = 0.038), high level of expression of Caspase-1mRNA (aOR 2.803, 95% CI 1.065–7.377, P = 0.037) and IL-18 cytokine (aOR 10.131, 95% CI 1.616–63.505, P = 0.013) had significant poor CD4^+^ T cell recovery.

**Conclusions:**

The baseline CD4^+^ T cell counts less than 100 cells/μL, high relative expression of Caspase-1 mRNA, and high expression of IL-18 cytokine are associated factors that affect the reconstruction of immune function.

## Introduction

Highly active anti-retroviral therapy (HAART) can reduce viral load and recover the number of CD4^+^ T cells, which promote immune reconstitution, prevent opportunistic infection, and improve the quality of life in HIV-1-infected patients. However, about 15–30% of HIV-1-infected patients, even after achieving viral suppression to undetectable levels, still fail to recover their CD4^+^ T cells after long-term regular HAART. This phenomenon is called immune reconstitution failure, and the patients are defined as immunological non-responders (INR) [1, 2]. Although Continuous immune activation and chronic inflammation in patients are suggested to play important roles in immune reconstitution after HAART treatment [3, 4], the mechanism for immune reconstitution failure after HAART treatment is still unclear.

Pyroptosis is an inflammatory programmed cell death, which is an important method of innate immunity against infection of HIV [5]. Due to abortive infection, caspase-1 is activated by CD4^+^ T cells through interferon-inducible protein 16 (IFI16). The activated caspase-1 cleaves the GSDMD protein and results in the pyroptosis of CD4^+^ T cells [6]. In addition, caspase-1 can also mediate the maturation and secretion of the pro-inflammatory cytokines IL-1β and IL-18, resulting in local chronic inflammation, and recruiting more immune cells to the sites of infection thus promoting more cell death [7, 8].

Some studies had found that even without HIV replication, the remaining over 95% of quiescent CD4^+^ T cell deaths were mainly caused by caspase-1-mediated pyroptosis [9, 10]. Meanwhile, Caspase-1 inhibitors could significantly prevent CD4^+^ T cells from further death [11]. Therefore, pyroptosis may result in increased death of CD4^+^ T cells, even without being infected by HIV, thereby affecting the reconstruction of immune function in HIV-1-infected patients.

To explore the clinical relevance between caspase-1- mediated cell pyroptosis and the immune reconstitution of HIV-1-infected patients, we evaluated the expression of caspase-1, GSDMD, IL-1β and IL-18 in peripheral blood of HIV-1-infected patients, and combined the clinical characteristics to find the associated factors that affect reconstitution of immune function.

## Materials and methods

### Study population

The study was enrolled at Wuming Hospital of Guangxi Medical University and Mashan People's Hospital. The study consists of 135 adult patients followed between 2007 and 2019. All participants agreed to participate in this study, and the ethics committee approved.

Inclusion criteria were as followed: ≥ 18 years old; received continuous treatment for at least 48 weeks after starting HAART; achieved and maintained virological suppression with virus load (VL) < 50 copies/mL or under the lower detectable limit. Exclusion criteria were as followed: VL ≥ 50 copies/mL or the VL detection had been interrupted during the treatment; drug resistance during HAART treatment.

Immunological non-responders group (INR) were defined as the last CD4^+^ T counts increased to less than 200 cells/μL after 48 weeks of treatment. The immunological responders group (IR) were defined as the last CD4^+^ T cell counts increased to more than 200 cells/μL after 48 weeks of treatment. In addition, HIV-1-infected patients with normal immune function were selected as the control group (NC), defined as CD4^+^ T cell counts remaining above 500 cells/μL during 48 weeks of treatment.

### Blood collection and separation

The whole blood was collected by venipuncture in Vacutainer tubes with or without containing EDTA, respectively. After 2 h later at room temperature, the whole blood without EDTA was centrifuged for 15 min at 1000 × g to separate the serum. The serum was aliquoted and stored at − 80 °C to avoid repeated freeze–thaw cycles.

### RNA extraction

The whole blood containing EDTA was added to Total RNA Extraction Reagent (9108, Takara, Japan) to extract RNA following the manufacturer’s recommended protocol. RNA quantification and purity were assessed using the NanoDrop 2000 spectrophotometer (ThermoFisher, US).

### Real-time quantification polymerase chain reaction (RT-qPCR amplification)

RNA was reversed transcripted to cDNA by PrimeScript™ RT reagent Kit with gDNA Eraser (RR047A, Takara, Japan). The cDNA was amplified by using GreenTB ® Premix Ex Taq™ II kit (RR820A, Takara, Japan). GAPDH was used as housekeeping genes to detect the relative expression of GSDMD and Caspase-1. Primer sequences were showed in Table 1.

**Table 1:** The primers sequences of target genes and reference gene

Gene	Sequence (5′–3′)
Caspase-1	
Forward	AGTGCAGGACAACCCAGCTATG
Reverse	CAAGACGTGTGCGGCTTGA
GSDMD	
Forward	TGAATGTGTACTCGCTGAGTGTGG
Reverse	CAGCTGCTGCAGGACTTTGTG
GAPDH	
Forward	TCTACTGGTTCAGCAGCCATCTTTA
Reverse	TGGTGAAGACGCCAGTGGA

*GSDMD* gasdermin D, *GADPH* glyceraldehyde-3-phosphate dehydrogenase

### Enzyme-linked immunosorbent assay (ELISA)

The expression of Caspase-1 and GSDMD in peripheral blood serum were quantified by Human Interleukin Caspase-1 ELISA Kit (CSB-E13025h CUSABIO, China) and Human Interleukin GSDMD ELISA Kit (BS-E6359H1, BOSHEN, China), respectively. The expression of IL-1β and IL-18 in peripheral blood serum were quantified by Human Interleukin 1β ELISA Kit (CSB-E08053H, CUSABIO, China) and Human Interleukin 18 ELISA Kit (CSB-E07450H, CUSABIO, China), respectively. The experimental operation was carried out strictly following the manufacturer’s instructions.

### Statistical processing

The continuous variables, except the age, were presented as means ± standard deviation (means ± SD). Levene method was used for the homogeneity of variance test. If P-value > 0.05, one-way ANOVA was performed for statistics, and then LSD method was used for pin-pair test. If P-value < 0.05, the variance was considered uneven, and then Welch method was used. Univariate analysis and then multivariate logistic regression models were applied to analyze the high-risk factors that affect the immune reconstitution between INR and IR. The two-sided P values < 0.05 were considered statistically significant. All analyses were performed using SPSS 22.0.0.1. Graphpad Prism 8.0.2. was used for mapping.

## Results

### Baseline characteristics of HIV-1-infected patients

A total of 135 participants who had been treated with ART for 48 weeks were recruited into the study. At 48 weeks after initiating ART, all participants had a suppressed viral load. The median age was 51 (30–79) years, including 86 males and 49 females. Among them, 57 patients with the last CD4^+^ T cell counts of 145.11 ± 36.43 cells/μL were classified as INR, whereas 56 patients with the last CD4^+^ T cell counts of 404.64 ± 122.41 cells/μL, were included in the IR group. Otherwise, 22 patients with the last CD4^+^ T cell counts of 727.91 ± 188.07 cells/μL were collected as the NC group. The demographic and clinical characterization data were shown in Table 2.

**Table 2:** Demographic and clinical characteristics of HIV-infected patients

	INR n = 57	IR n = 56	NC n = 22
Gender			
Male	78.9% (45)	55.4% (31)	45.5% (10)
Female	21.1% (12)	44.6% (25)	54.5% (12)
Age (years)			
21–40	12.3% (7)	23.2% (13)	18.2% (4)
41–60	52.6% (30)	66.1% (37)	54.5% (12)
61–80	35.1% (20)	10.7% (6)	27.3% (6)
BMI	19.39 ± 4.45	20.54 ± 2.26	20.06 ± 6.24
HIV transmission category			
Heterosexual	96.5% (55)	98.2% (55)	100% (22)
Homosexual	3.5% (2)	0.0%	0.0%
Injection drug user	0.0%	1.8% (1)	0.0%
Regimen			
TDF + 3TC + EFV	61.4% (35)	44.6% (25)	68.2% (15)
AZT/3TC + EFV	19.3% (11)	23.2% (13)	18.2% (4)
Other	19.3% (11)	32.2% (18)	10.7% (3)
Baseline CD4^+^ T Counts (cells/μL)	92.91 ± 94.53	115.22 ± 79.29	561 ± 199.91
Baseline CD8^+^ T Counts (cells/μL)	785.41 ± 462.31	728.01 ± 437.47	1165.41 ± 488.1
Baseline CD4/CD8 ratio	0.13 ± 0.15	0.18 ± 0.15	0.53 ± 0.18
Baseline WBC (× 10^9^ / L)	5.04 ± 1.61	5.03 ± 1.86	7.65 ± 3.62
Last CD4^+^ T Counts (cells/μL)	145.11 ± 36.43	404.64 ± 122.41	727.91 ± 188.07

### The expression of pyroptosis is increased in peripheral blood of patients with immunological non-response

**Fig. 1:**
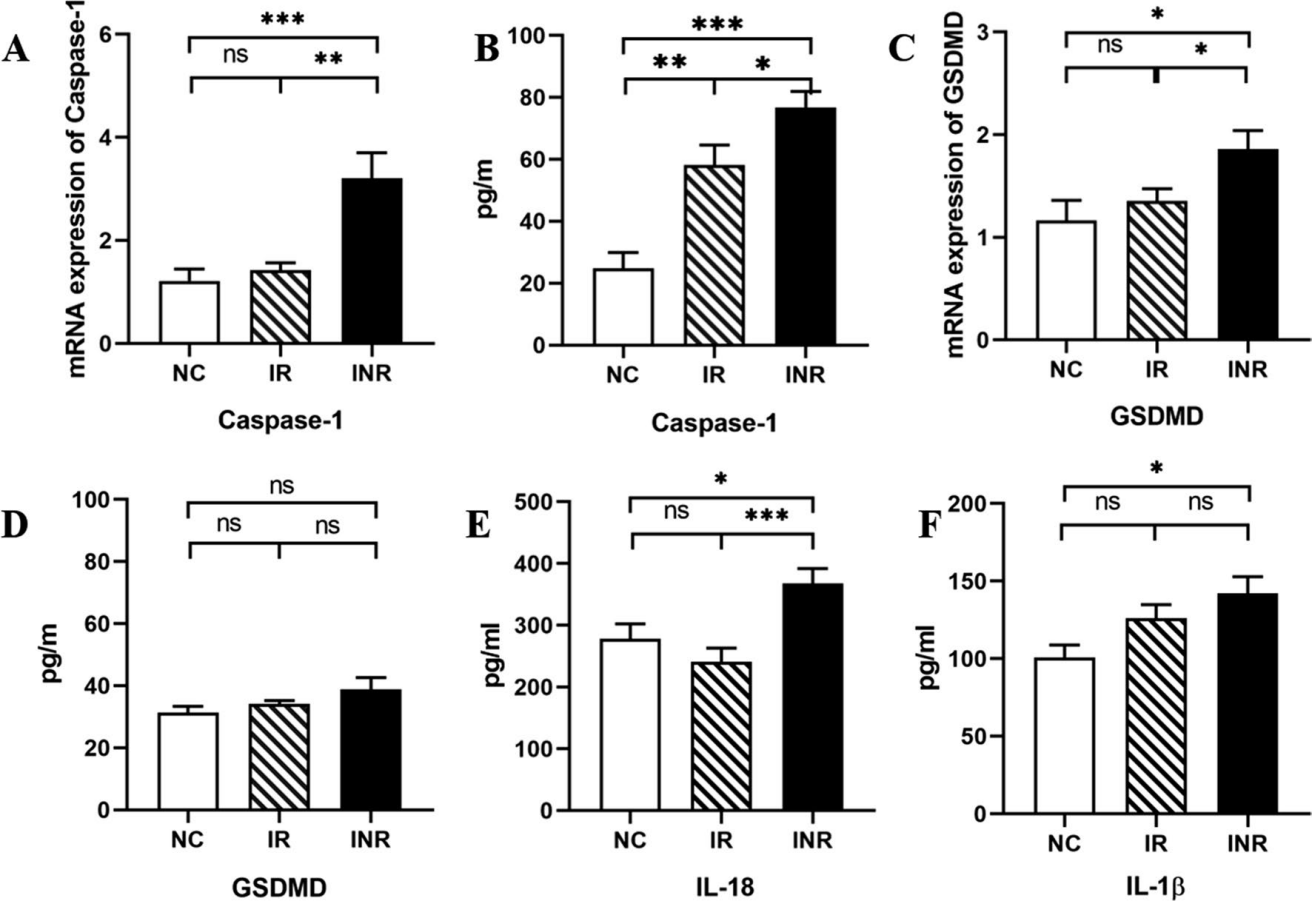
The expression of pyroptosis in peripheral blood of HIV-1-infected patients. **A**, **C** The mRNA expression of Caspase-1 and GSDMD in NC, IR and INR. **B**, **D**, **E**, **F** The cytokine concentrations of Caspase-1, GSDMD, IL-1β and IL-18 in group NC, IR and INR. *P < 0.05; **P < 0.01; ****P < 0.001; *ns* no significant differences

GSDMD is an executor of pyroptosis [12–14]. CD4^+^ T cell death infected by HIV-1 is mainly caused by caspase-1-mediated pyroptosis [15]. We evaluated the differential expression of caspase-1-mediated pyroptosis in three groups. Compared with the IR and the NC, the INR showed higher expression of Caspase-1 mRNA (Fig. 1A) and GSDMD mRNA (Fig. 1C) in peripheral blood. No significant differences were observed in the relative expression of the GSDMD mRNA and Caspase-1 mRNA between the IR and the NC. In peripheral serum, the INR showed higher cytokine expression of Caspase-1 (Fig. 1B). However, we did not observe differences of GSDMD in the three groups (Fig. 1D).

### Higher levels of IL-18 in plasma in the INR

We further investigated the expression of IL-18 and IL-1β in peripheral serum. Results indicated that the expression of IL-18 in the INR group increased significantly (Fig. 1C), which was statistically different compared to the IR and the NC. However, we did not observe differences in the IL-1β between the INR and the IR (Fig. 1D). The difference approached but did not reach statistical significance.

### Pyroptosis is independently associated with CD4^+^ T cell recover

In order to further determine the association between the expression of cell pyroptosis productions and the recovery of CD4^+^ T cells in blood, logistic regression analysis was performed. Firstly, results of univariate regression analysis indicated a significant association between the last CD4^+^ T cell counts and the gender (OR 3.024, 95% CI 1.323–6.911, P = 0.009), age (OR 4.505, 95% CI 1.646–12.324, P = 0.003), baseline CD4^+^ T cell counts < 100 cells/mm^3^ (OR 3.636, 95% CI 1.666–7.939, P = 0.001), the expression of GSDMD mRNA (OR 1.636, 95% CI 1.016–2.633, P = 0.043), Caspase-1 mRNA (OR 1.660, 95% CI 1.158–2.379, P = 0.006), Caspase-1 cytokine (OR 2.716, 95% CI 1.019–7.235, P = 0.046) and IL-18 cytokines (OR 3.883, 95% CI 1.692–8.907, P = 0.001) in the INR.

After adjusting for confounders from the univariate analysis, multivariate Logistic regression indicated that the last CD4^+^ T cell counts were independently associated with the baseline CD4^+^ T cell counts less than 100 cells/μL (aOR 7.051, 95% CI 1.115–44.592, P = 0.038), the relative expression of Caspase-1mRNA (aOR 2.803, 95% CI 1.065–7.377, P = 0.037) and high IL-18 cytokine expression (aOR 10.131, 95% CI 1.616–63.505, P = 0.013) in the INR. These factors will increase the risk of immune reconstitution failure, and were the independent factors associated with immune reconstruction in HIV-1-infected patients (Table 3).

**Table 3:** Factors associated with CD4^+^ T cells recovery in HIV-1-infected patients

	Univariable analysis		Multivariable analysis	
	OR (95% CI)	*P*	a0R (95% CI)	*P*
Gender				
Male	1.0		1.0	
Female	3.024 (1.323, 6.911)	0.009	0.972 (0.13, 7.28)	0.978
Age (years)				
≤ 60	1.0		1.0	
> 60	4.505 (1.646, 12.324)	0.003	3.888 (0.465, 32.519)	0.210
BMI (kg/m^2^)	0.887 (0.742, 1.06)	0.187		
Transmission category				
Heterosexual	1.0			
Others	0.988 (0.196, 4.99)	0.988		
Initial HAART regimen				
TDF + 3TC + EFV	1.0			
AZT/3TC + EFV	2.291 (0.923, 5.685)	0.074		
Others	1.385 (0.461, 4.155)	0.562		
Baseline CD4^+^ T cell Counts (cells/μL)				
≥ 100 cells/μL	1.0		1.0	
< 100 cells/μL	3.636 (1.666, 7.939)	0.001	7.051 (1.115, 44.592)	0.038
Baseline CD8^+^ T cell counts^a^	1.024 (0.91, 1.152)	0.697		
Baseline CD4/CD8 ratio	0.11 (0.003, 3.497)	0.211		
Baseline WBC(× 10^9^/L)^b^	0.94 (0.736, 1.201)	0.619		
The relative expression of GSDMD mRNA^c^	1.636 (1.016, 2.633)	0.043	2.49 (0.779, 7.959)	0.124
The relative expression of Caspase-1 mRNA ^c^	1.66 (1.158, 2.379)	0.006	2.803 (1.065, 7.377)	0.037
The expression of GSDMD^d^	1.541 (0.578, 4.112)	0.388		
The expression of Caspase-1^e^	2.716 (1.019, 7.235)	0.046	1.846 (0.291, 11.691)	0.515
The expression of IL-18 cytokine^f^	3.883 (1.692, 8.907)	0.001	10.131 (1.616, 63.505)	0.013
The expression of IL-1β cytokine^g^	1.151 (0.846, 1.565)	0.370		

Per 1-year increase in duration

*OR* odds ratio; *CI* confidence interval; *BMI* body mass index; *aOR* adjust odds ratio

^a^Per 100 cells/μL increase

^b^Per 1 × 10^9^ cells/μL increase

^c^Per 1 log increase

^d^Per 30 pg/mL increase

^e^Per 50 pg/mL increase

^f^Per 300 pg/mL increase

g Per 100 pg/mL increase

## Discussion

Due to the lower number of CD4^+^ T cells, INR were at greater risk of poor long-term prognoses such as disease progression, opportunistic infections and AIDS-related mortality despite viral suppression after years of continuous HAART [16]. However, the mechanisms of immune reconstitution failure after HAART remained unclear, and there is also no effective treatment. Therefore, looking for the marker associated with poor immune reconstitution is important to improve the prognosis for HIV-1-infected patients.

Pyroptosis was the body’s immune response against important pathogen infection [8, 17–19]. Previous study had shown [15] that 95% of CD4^+^ T cell death is caused by caspase-1 mediated pyroptosis, while only 5% of quiescent CD4^+^ T cells died due to the replication of HIV-1. Bandera [20] found that the expression of NLRP3 inflammasome and Caspase-1 was increased in PBMC cells stimulated in vitro from the patients with immune reconstitution failure, compared with patients with full immune reconstitution. Pyroptosis was overactivated in patients with immune reconstitution failure. Our study found that expression of Caspase-1 and downstream target genes GSDMD were significantly increased in peripheral blood of INR. Interestingly, there was no significant difference between IR and NC. Our finding showed that Caspase-1 mediated pyroptosis was associated with the failure of immune function reconstruction in HIV-1-infected patients. However, we did not observe significant differences in the expression of the GSDMD in the peripheral serum**.** Caspase-1 specifically cleaved the linker between the amino-terminal gasdermin-N and carboxy-terminal gasdermin-C domains in GSDMD, resulting in the gasdermin-N domain triggering pyroptosis [14]. Lorenzo Sborgi [21] found that GSDMD processing correlated with caspase-1 activation and was detectable in the cell supernatant in vitro. However, we found it was highly challenging to reliably quantify the cleaved GSDMD in the blood sample, since upon GSDMD cleavage cells lyse and was degraded rapidly. Song [22] found that the expression of Caspase-1 increased rapidly and then decreased within a short period of time in the high CD4^+^ T cell counts group during the early stage of HIV-1 infection. On the contrary, the expression of Caspase-1 increased significantly in the low-level CD4^+^ T cell counts group after one year of HIV-1 infection. This finding indicated that pyroptosis, as an innate immune response, promotes virus clearance to facilitate immune function reconstruction in the early stage of HIV infection, whereas overactivation pyroptosis triggered the increasing of CD4^+^ T cell death at a later stage of infection, resulting in immune reconstruction failure. Furthermore, from the multivariate analysis, we also found that the expression of Caspase-1 was significantly associated with immune reconstitution, which could be used as a convenient immune-associated factor in clinical practice.

Pyroptosis was mainly manifested as the continuous expansion of the cell membrane until it ruptures, and then the release of a large amount of intracellular material triggers a strong inflammatory response [23]. Caspase-1 cleaved the precursors of IL-18 and IL-1β into active IL-18 and IL-1β, which were released into the extracellular after activation. Activated IL-18 and IL-1β can recruit and activate other immune cells, and induce the synthesis of IL-6, chemokines, and adhesion molecules, thereby amplifying the local and systemic inflammatory response during the process of pyroptosis. Some experiments in vitro have proved [24, 25] that HIV could induce microglia to express NLRP3 inflammasomes and increase the expression levels of Caspase-1 and IL-1β, resulting in the inflammation of the central nervous system. Feria [26] found that the expression levels of Caspase-1, IL-1β and IL-18 were significantly up-regulated and associated with CD4^+^ T cell counts in peripheral blood mononuclear cells (PBMC) from patients with HIV disease progression. Andrade [27] found that Both IL-18 rs187238 G allele and GG genotype were closely related to the recovery of immune reconstitution function. Our finding was consistent with these studies that IL-18 was significantly up-regulated and resulted in chronic inflammation, involved in the occurrence and development of immune reconstitution failure. However, we did not find a statistically significant difference in the expression of IL-1β between the two groups of patients. This may be related to the small sample size in this study, and needs to be further studied.

Previous studies showed that older age [28, 29], male [30–32], and lower baseline CD4^+^ T cell counts [33–36] were risk factors for CD4^+^ T cell counts recovery after HAART initiation, but our study showed that low baseline CD4^+^ T cell counts was the main risk factor affecting immune reconstitution, after adjusting for confounders from the univariate model. A retrospective study in Australia [33] showed that HIV-1-infected patients who had sustained virological suppression for more than 5 years after receiving HAART treatment, had lower baseline CD4^+^ T cells percentage and longer time to reach a plateau CD4^+^ T cell counts, as well as related to a lower plateau CD4^+^ T cell counts. Another Johns Hopkins cohort study found that [35] patients with a lower baseline CD4^+^ T cell counts achieved a lower plateau CD4^+^ T cell counts, and only patients with baseline CD4^+^ T cell counts > 350 cells/μL could return to a normal CD4^+^ T cell counts. This suggests that the baseline CD4^+^ T cell counts correlates with the effect of immune function reconstruction and can be used as an associated factor of INR.

This study also had certain limitations. Firstly, we only collected cross-section data at a short duration of follow-up, so it could not evaluate the dynamic evolution between CD4^+^ T cell counts recovery and the expression in pyroptosis of peripheral blood under the long-term HAART. Secondly, our result did not explain the specific mechanism of pyroptosis in patients with poor immune reconstitution, which need further investigation. Nevertheless, our finding provides a novel insight intothe new immune-associated factors in INR.

## Conclusion

Our findings showed that. Caspase-1 and IL-18 can be used as important predictors for immune function recovery, as well as baseline CD4^+^ T cell counts less than 100 cells/μL.

## Data Availability

The datasets used and/or analysed during the current study are available from the corresponding author on reasonable request.

## Abbreviations

HIV: Human immunodeficiency virus
HAART: Highly active anti-retroviral therapy
INR: Immunological non-responders
IR: Immunological responders
GSDMD: Gasdermin D
IFI16: Interferon-inducible protein 16
BMI: Body mass index
OR: Odds ratio
CI: Confdence interval
